# Specific profiles of new-onset vs. non-inaugural status epilepticus: From diagnosis to 1-year outcome

**DOI:** 10.3389/fneur.2023.1101370

**Published:** 2023-02-03

**Authors:** Marie Benaiteau, Luc Valton, Ludovic Gardy, Marie Denuelle, Rachel Debs, Valentin Wucher, Florence Rulquin, Emmanuel J. Barbeau, Fabrice Bonneville, Jérémie Pariente, Jonathan Curot

**Affiliations:** ^1^French Reference Center on Paraneoplastic Neurological Syndromes and Autoimmune Encephalitis, University Hospital of Lyon HCL, Lyon, France; ^2^Neurology Department, Toulouse University Hospital, Toulouse, France; ^3^Brain and Cognition Research Center (CerCo), French National Scientific Research Center, UMR5549, Toulouse, France; ^4^Synaptopathies and Autoantibodies (SynatAc) Team, NeuroMyoGene-MeLis Institute, INSERM U1314/CNRS UMR 5284, University of Lyon, Lyon, France; ^5^Faculty of Health, University of Toulouse-Paul Sabatier, Toulouse, France; ^6^INSERM, U1214, Toulouse Neuro Imaging Center (ToNIC), Toulouse, France; ^7^Neuroradiology Department, Toulouse University Hospital, Toulouse, France

**Keywords:** status epilepticus, new-onset status epilepticus, new-onset refractory status epilepticus (NORSE), peri-ictal MRI abnormalities, outcome, epilepsy, refractory status epilepticus (RSE)

## Abstract

While new-onset status epilepticus (NOSE) is a harbinger of chronic epilepsy, prospective medical data are sparse in terms of specifying whether the evolution of status epilepticus (SE) and seizure expression in NOSE resembles what occurs in patients who have already been diagnosed with epilepsy [non-inaugural SE (NISE)] in all aspects apart from its inaugural nature. The aim of this study was to compare the clinical, MRI, and EEG features that could distinguish NOSE from NISE. We conducted a prospective monocentric study in which all patients ≥18 years admitted for SE over a 6-month period were included. A total of 109 patients (63 NISE and 46 NOSE cases) were included. Despite similar modified Rankin scores before SE, several aspects of the clinical history distinguished NOSE from NISE patients. NOSE patients were older and frequently had neurological comorbidity and preexisting cognitive decline, but they had a similar prevalence of alcohol consumption to NISE patients. NOSE and NISE evolve in the same proportions as refractory SE (62.5% NOSE, 61% NISE) and share common features such as the same incidence (33% NOSE, 42% NISE, and *p* = 0.53) and volumes of peri-ictal abnormalities on MRI. However, in NOSE patients, we observed greater non-convulsive semiology (21.7% NOSE, 6% NISE, and *p* = 0.02), more periodic lateral discharges on EEG (*p* = 0.004), later diagnosis, and higher severity according to the STESS and EMSE scales (*p* < 0.0001). Mortality occurred in 32.6% of NOSE patients and 21% of NISE patients at 1 year (*p* = 0.19), but with different causes of death occurring at different time points: more early deaths directly linked to SE at 1 month occurred in the NOSE group, while there were more remote deaths linked to causal brain lesions in the NISE group at final follow-up. In survivors, 43.6% of the NOSE cases developed into epilepsy. Despite acute causal brain lesions, the novelty related to its inaugural nature is still too often associated with a delay in diagnosing SE and a poorer outcome, which justifies the need to more clearly specify the various types of SE to constantly raise awareness among clinicians. These results highlight the relevance of including novelty-related criteria, clinical history, and temporality of occurrence in the nosology of SE.

## 1. Introduction

It is interesting to note that recent acronyms and definitions of status epilepticus (SE) include the following references to time and the novelty of occurrence: *new-onset* status epileptic (NOSE) (1), *new-onset* refractory SE (NORSE) (2, 3), *late-onset* absence SE (4), and *subacute* encephalopathy syndrome in alcoholics (SESA) (5). Could the mode of onset, novelty, and temporal context be key to understanding SE? What makes NOSE a specific clinically relevant pathological entity that is distinguishable from other types of SE, i.e., non-inaugural SE that occurs in patients with epilepsy (NISE)?

Only recently have the temporality of onset and novelty become an integral part of the definitions of SE. Since the pioneer definitions of SE (4), many “mechanistic” or “operational” definitions of SE have been proposed (4, 6–10). Until now, they have been exclusively based on semiology (convulsive or non-convulsive, generalized or focal, etc.). The temporal dimension has long been considered regarding the duration of SE but not in the context of its onset. Operationally, NOSE is defined as “prolonged seizures lasting *more than 5 min* or the presence of recurrent seizures without return to baseline in between *in patients with no previous history of epilepsy*” (11). However, this definition remains non-consensual. SE nosology is constantly evolving due to the clinical heterogeneity of its semiology, a complex poorly understood pathophysiology (12, 13), multiple etiologies (14), and difficulties conducting prospective studies.

Clarifying the definition of NOSE is essential as the incidence is significant. Approximately half of all adult cases of SE (up to 59%) are inaugural in non-epileptic patients (15–20). The incidence of NOSE was found to be 16.3/100,000 to 36/100,000 adults per year depending on the cohort and whether or not the new ILAE 2015 definition and classification of SE was taken into account (21, 22). However, despite the incidence, knowledge of the clinical, EEG, and MRI spectrum of NOSE in adults is mostly based on retrospective data (11, 20, 23–25).

One of the few consensual elements concerning NOSE is a poor prognosis and a possible progression to refractory SE (i.e., NORSE) (24, 26–28). Mortality in 1 month is 20–61% depending on the cohort. Factors of poor prognosis are the age of the patient [especially over 65 years (11)], etiology, and the duration of the SE (15–17, 23, 24, 29, 30). Tracheal intubation and co-infections are additional factors of adverse outcomes (23).

In survivors, a poor prognosis for NOSE also suggests the onset of a chronic illness. More than 58% of survivors may experience seizures, mainly related to acute or progressive brain injury, the duration of SE (significant threshold at 24 h) being the only independent predictor of the development of chronic epilepsy after SE (27). Paradoxically, some series also showed that progression to NORSE had no influence on functional outcome or mortality at the last follow-up, while SE semiology (non-convulsive vs. convulsive and loss of consciousness) or age above or equal to 65 did not predict progression to NORSE (11).

If NOSE is a precursor to chronic epilepsy, it could be hypothesized that its presentation resembles NISE in all aspects apart from its inaugural nature. However, the medical literature is unable to demonstrate this. None of the studies cited above investigated the discriminating features between NOSE and NISE. In addition, little information is available on the paraclinical aspects associated with NOSE, and the most recent publications frequently focused on the refractory subtype of these *de novo* SE (2, 3, 31).

Although it is now well-established that NOSE can develop into epilepsy, to our knowledge, there is no prospective trial that compares the clinical, MRI, and EEG patterns that may distinguish NOSE from NISE. Does the mechanism that leads to epilepsy result in a specific clinical pattern of SE? Are there imaging and electrophysiological criteria that distinguish NOSE from NISE? Do NOSE and NISE progress similarly and have the same prognosis?

To clarify these questions, we conducted a prospective monocentric study to multimodally compare NOSE and NISE at baseline (before SE), during SE, and at follow-up in 1, 3, and 12 months. The aims of this study were (1) to compare clinical and paraclinical (brain imagery and electrophysiological recordings) features of NOSE (including NORSE) and NISE; (2) to study the outcome of SE at 1, 3, and 12 months as well as the prognostic factors; and (3) more specifically to analyze peri-ictal MRI abnormalities. We hypothesized that NOSE and NISE each have their own specificities, particularly in terms of outcomes. We hoped to identify new markers for positive diagnosis and the prognosis of inaugural SE.

## 2. Methods

### 2.1. Design and population

**Figure 1:**
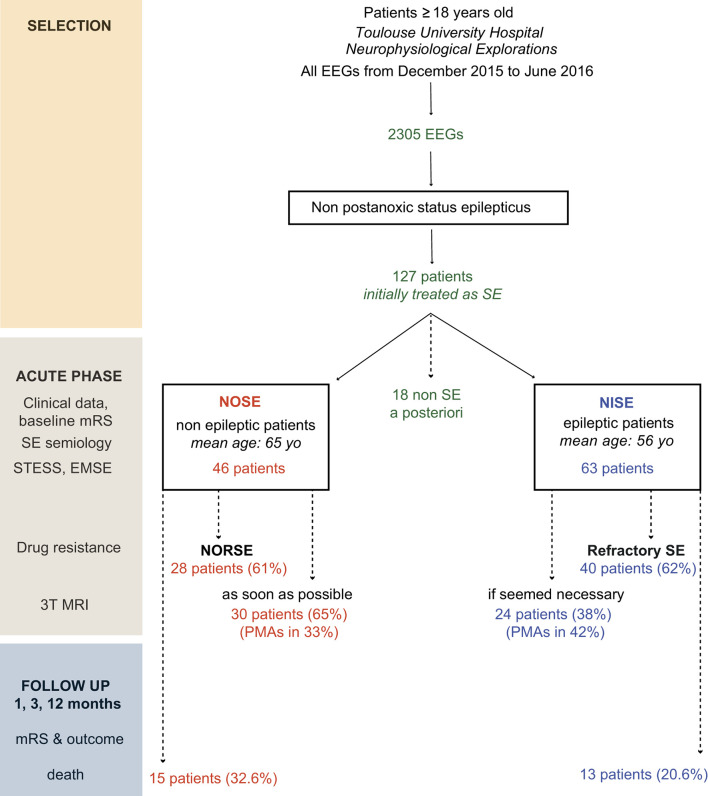
Study design, selection of patients, and SE classification. Five patients were secondarily excluded from the NOSE group (1 with myoclonus of the left upper limb secondary to spinal cord ischemia, 1 with vigilance fluctuations due to a post-traumatic brainstem lesion, 1 with a reactive coma and abnormal movements secondary to severe intra-parenchymal hemorrhage, 1 with psychomotor agitation and vagal discomfort with the loss of consciousness due to pain, and 1 with a first psychogenic non-epileptic status). A total of 13 patients were secondarily excluded from the NISE group (6 with a psychogenic non-epileptic status, 3 with serial seizures but complete clinical recovery between seizures, 3 with a prolonged post-ictal deficit and/or post-ictal agitation, and 1 with chronic meningitis on ventriculoperitoneal shunt, abnormal eye movements, and intracranial hypertension with no argument for seizures).

Our work is a prospective, observational, descriptive, single-center study (Figure 1). We collected clinical, neuroradiological, and electrophysiological data for each patient admitted consecutively to Toulouse University Hospital from December 2015 to June 2016 (1) for SE and (2) SE not clinically diagnosed immediately on admission but subsequent to the first EEG. To select these patients, all EEGs performed during this period and all requests for EEGs for SE were screened. The inclusion criteria were a diagnosis of SE confirmed by a neurologist, exclusion of post-anoxic SE, age of 18 years and older, and non-refusal to participate in the study. The use of the data in our study was approved by the Regional Ethics Committee at Toulouse University Hospital (CPP Sud-Ouest no. 04-1215).

### 2.2. SE definitions and classifications

Epilepsy is defined as a lasting predisposition to generate seizures and the cognitive, behavioral, psychological, and social consequences of this condition (32). NOSE was defined as the occurrence in patients without a history of SE or uncured epilepsy (cured epilepsy is the absence of seizures in the absence of treatment for more than 5 years). The following functional and semiological definitions were used for either NOSE or NISE.

The definitions used for *generalized* and *focal convulsive* SE were (10, 21, 33) the occurrence of at least two epileptic seizures in a short interval without complete recovery of a stable neurological status between seizures; ictal clinical or electrical activity lasting 5 min or more for generalized seizures and 10 min or more for focal seizures; serial seizures; and seizures followed by a coma or persistent confusion.

The definition used for *non-convulsive* SE (NCSE) was based on the Salzburg consensus criteria (34, 35). We considered that an NCSE was *certain* if there was an association with an acute qualitative or quantitative alteration of consciousness without prominent motor symptoms *or* persistent after a clinical seizure with motor manifestation, not otherwise explained, *and* electrical confirmation by EEG (pattern of epileptiform discharges at a frequency >2.5 Hz present for more than 10 recorded seconds). When epileptiform discharges were present <2.5 Hz in the worst 10-s epoch or there was no epileptiform discharge but only continuous rhythmic delta-theta activity >0.5 Hz, the following secondary criteria were fulfilled: (a) typical spatio-temporal evolution or (b) subtle clinical ictal phenomena were present during the patterns, or (c) a clear clinical *and* EEG improvement after intravenous administration of an appropriately chosen anti-seizure medication (ASM) was documented.

### 2.3. Clinical data

In the acute phase, we collected the following data: age, gender, personal history, modified Rankin score (mRS) before SE (at baseline), medications, clinical symptoms of the seizures observed *during* SE, and *post-ictal* deficit. Severe and life-threatening complications were specified: respiratory distress, including infectious pneumonia, hemodynamic instability (systolic blood pressure <90 mmHg; the need for vasoamines), and traumatic complications.

SE duration was calculated or estimated through a combination of clinical and EEG data. SE was considered refractory if it persisted 30 min after the introduction of a first- and second-line ASM (9, 36) and super-refractory if it persisted at least 24 h after the start of general anesthesia (37, 38).

The etiologies and/or contributing factors of SE were classified according to the ILAE Task Force definition: acute etiologies, sequelae or old structural abnormalities, progressive etiologies, known epileptic syndromes, and unknown etiologies (21, 39). SE severity was rated by two scales, namely, the Status Epilepticus Severity Score (STESS) (40) and the Epidemiology-Based Mortality Score in Status Epilepticus (EMSE) (41).

The cognitive status of non-epileptic patients before SE was estimated using the long version (a 26-item questionnaire) of the IQ CODE (42). In the literature, the threshold chosen for diagnosing dementia is 3.4/5 (43).

The chronology of follow-up was 1, 3, and 12 months to detect early, medium-term, and late complications, respectively (mRS, onset of recurrent epileptic seizures or new SE, and death). Cognitive complaint and focal neurological deficit were specified at 3 months.

### 2.4. Electrophysiological data

Scalp-EEGs (9–21 surface electrodes, 256 Hz sampling rate) combined with video and ECG were recorded with the Deltamed system (Natus Medical Incorporated). At least 20 min were recorded for each patient (12 patients were monitored for several hours). The time period between the first EEG and the onset of symptoms was noted. EEG recordings were analyzed by clinical electrophysiologists (JC, MB, MD, RD, and LV). EEGs were classified as “normal,” “sedation EEG,” “ictal,” or “post-ictal” EEG. Epileptic activities were divided into “periodic discharges,” “rhythmic delta discharges,” and “paroxysmal abnormalities” (i.e., spikes, polyspikes, and spikes-and-waves) (21).

### 2.5. MRI

We used two 3T imagers (Magnetom Skyra, Siemens Healthcare and Achieva, Philips Medical System) in the clinical neuroradiology department. An MRI was performed urgently as soon as the patient's condition allowed, ideally within 72 h of the diagnosis of NOSE and if considered necessary for the care of patients with NISE. A minimum of the following sequences was performed: DWI, ADC mapping, fluid-attenuated inversion recovery (FLAIR), T1 with gadolinium, and gradient-recalled echo T2^*^. The presence of PMAs in DWI and FLAIR sequences, the volume of PMAs in DWI, the presence and type of old cerebral lesions, and SE etiology were analyzed by a trained neuroradiologist and neurologist (FB and MB). When other lesions (peritumoral edema, gliosis, acute stroke, etc.) could explain the abnormalities in DWI and FLAIR, these were not considered as PMAs.

We semi-automatically quantified the PMA volume in DWI using OLEA software in a one-shot analysis with manual correction [Olea Sphere^®^ version 2.3, cutting thickness of 3 or 4 mm, technique validated for ischemic stroke (44)]. The PMA volume was estimated using the average of three different segmentations for each patient. The standard zones of the magnetic susceptibility artifact were systematically trimmed.

If an MRI control was required, it was scheduled on the same 3T machines within 3 months of the SE.

### 2.6. Statistical analyses

To compare the NOSE and NISE groups, we used the chi-square test for qualitative variables, except when the theoretical numbers were <5, in which case, Fisher's test was used. To compare quantitative variables, we used the Wilcoxon test. To limit the risk of type-1 errors associated with multiple comparisons, we corrected the alpha values using the Bonferroni method. The alpha values to be considered are indicated below the figures or tables (in general, the alpha value = 0.05/90). Due to the inherent heterogeneity of clinical data and the multiple comparisons made, we considered “tendencies” for *p* < 0.05 but above the corrected threshold. We only considered significant *p*-values below the corrected alpha values. For *post-hoc* analyses, Tukey's test was used (*p*-values automatically adjusted for multiple comparisons).

To study the impact on outcome (mRS) at different timestamps for predictive factors such as the presence or absence of PMA, drug resistance, or status epilepticus, we performed multiple linear regressions. We used linear mixed-effects models in which the variable “patient” was considered a random effect. The variables “measurement time” (baseline, first month, third month, and twelfth month), drug resistance (1/0), with or without PMA (1/0), and new-onset status epilepticus (1/0) were considered as fixed effects. Finally, Pearson correlations were performed. The corrected *p*-value was considered for the significance threshold for linear mixed-effect models, and the Pearson correlation was 0.017 (0.05/3).

## 3. Results

### 3.1. A large prospective cohort of SE including NOSE and NISE

During the 6 months, 2,305 EEGs were performed, allowing the inclusion of 127 patients who had been treated for SE. A total of 18 patients initially considered as having SE were excluded after revision of the diagnosis *a posteriori*: five patients from the NOSE group and 13 epileptic patients from the NISE group. Therefore, 109 patients (46 NOSE and 63 NISE cases) were finally included (Figure 1).

### 3.2. Clinical history distinguishing NOSE and NISE patients

Clinical data are presented in Table 1. Despite a similar level of autonomy on the mRS before SE, patients experiencing NOSE tended to be older (*p* < 0.01, alpha = 0.0006). The same proportion of excessive alcohol consumption, psychiatric history, and use of psychotropic drugs was found in both groups. Alcohol abuse or dependence was directly involved in 5 NOSE patients and 6 NISE patients. The IQ code before SE was obtained for the NOSE group only: 28/46 (61%) patients had a score of ≥3.4/5, which is above the threshold indicating significant cognitive impairment that affects autonomy in daily life. All patients in the NISE group had been on ASM (median = 1, min = 1, max = 5). In total, 24 of 57 NISE patients (42%) had a history of SE (data are lacking for six subjects). Epilepsy was considered stabilized (seizure-free patients) for 38 of 59 patients before the onset of SE (data are lacking for four patients).

**Table 1:** Clinical characteristics of NOSE and NISE patients.

	**Total SE**	**NOSE**	**NISE**	***p***
	***n*** = **109**	***n*** = **46**	***n*** = **63**	
Female gender	52 (47.7%)	21 (45%)	31 (49%)	0.71
Age (years)	60	65.7 (22–101)	56.3 (15–90)	0.01^∧^
Neurologic history	65 (60%)	24 (52%)	41 (65%)	0.49
Cured epilepsy	3	3 (6.5%)	0	0.14
Severe cranial trauma	29	7 (15%)	12 (19%)	0.58
Stroke	23	15 (32.6%)	8 (12.7%)	0.01
Cerebral hemorrhage	11	2 (4.3%)	9 (14 %)	0.11^#^
Cerebral tumors	14	2 (4.3%)	12 (19%)	0.02
CNS infections	3	0	3 (4.7%)	0.26^#^
Psychiatric history	24 (22%)	10 (21.7%)	14 (22%)	0.95
Alcohol abuse	24 (22%)	8 (17.4%)	16 (25%)	0.32
Daily use of psychotropic drugs	44 (40%)	17 (37%)	27 (43%)	0.54
IQ code ≥ 3.4/5	–	28 (60.9%)	–	
Modified Rankin Scale before SE				
0–1	38	18 (39%)	20 (31.7%)	0.55
2–3	61	22 (47.8)	39 (62%)	0.2
4–5	10	6 (13%)	4 (6.3%)	0.39
SE type				
Generalized convulsive	39 (35.5%)	16 (34.7%)	23 (36.5%)	0.85
Focal convulsive	44 (40%)	20 (43.5%)	24 (38%)	0.57
Non-convulsive	26 (24%)	10 (21.7%)	16 (25.5%)	0.66
Secondary non-convulsive	14 (12.8%)	10 (21.7%)	4 (6%)	0.02
Secondary generalized convulsive	29 (26.6%)	14 (30.4%)	15 (24%)	0.44
Median time between SE and diagnosis (min-max)	30 min (0–15 d)	60 min (0–15 d)	10 min (0–30 h)	0.006^∧^
Median time between SE and therapeutic care (min-max)	90 min (0–15 d)	90 min (10 min–15 d)	60 min (5 min–30 h)	0.09^∧^
Average number of ASMs needed to stop SE (min-max)	3 (1–7)	3 (1–7)	2.7 (1–6)	0.24^∧^
ASMs used for SE				
Benzodiazepines	95 (87%)	41 (89%)	54 (85.7%)	0.81
Phosphenytoin	45 (41%)	25 (54%)	20 (31.7%)	0.03
Broad spectrum ASM	68 (62%)	27 (59%)	41 (65%)	0.63
Narcotics	34 (32%)	19 (41.3%)	15 (23.8%)	0.08
Severe complications	42 (38.5%)	23 (50%)	19 (30%)	0.057
Respiratory	30 (27.5%)	15 (32.6%)	15 (24%)	0.42
Hemodynamic	20 (18%)	13 (28.2%)	7 (11%)	0.04
Trauma-related	13 (12%)	6 (13%)	7 (11%)	0.99
Intensive care	39 (35.5%)	16 (35%)	23 (36%)	0.85
Ventilated-intubated	26 (24%)	13 (28%)	13 (20%)	0.49
Post-ictal vigilance disorders	72 (66%)	34 (73.9%)	38 (60%)	0.2
Focal post-ictal neurological deficit	76 (70%)	37 (80.4%)	39 (62%)	0.06
STESS median	3/6	4/6	2/6	<0.0001
Poor outcome threshold ≥ 3/6	59 (54 %)	36 (78%)	23 (36.5%)	
EMSE median	58/255	67/255	50/255	<0.0001
Poor outcome threshold ≥ 64/255	47 (43 %)	29 (63%)	18 (28.5%)	
Average SE duration (min-max)	40 h (15 min–41 d)	62 h (15 min–41 d)	23 h (30 min–8 d)	0.33
Pharmacoresistance				
Refractory SE	68 (62%)	28 (61%)	40 (62.5%)	0.93
Super-refractory SE	9 (8%)	5 (11%)	4 (6%)	0.49^#^
SE etiologies				
Acute etiologies	25 (23%)	20 (43.5%)	5 (7.9%)	<0.0001
Acute cerebral injury on MRI	13 (12%)	10 (21.7%)	3 (4.8%)	0.01
Remote etiologies	41 (37.7%)	14 (30.4%)	27 (42.9%)	
Without acute trigger	16 (14.7%)	7 (15.2%)	9 (14.3%)	0.73
Remote with acute trigger	25 (23 %)	7 (15.2%)	18 (28.6%)	0.09
Progressive etiologies	17 (15.6%)	7 (15.2%)	10 (15.8%)	0.93
Defined electroclinical syndromes	22 (20%)	1 (2.1%)	21 (33.3%)	<0.0001
With acute trigger	12 (11%)	1 (2.1%)	11 (17.4%)	0.01
Unknown/cryptogenic	4 (3.7%)	4 (8.7%)	0	0.029^#^
Mortality				
At 1 month	11 (10%)	7 (15.2%)	4 (6.4)	0.20^#^
At 3 months	16 (14.7%)	11 (24%)	5 (8%)	0.02
At 1 year	28 (25.7%)	15 (32.6)	13 (20.6%)	0.19

Psychotropic drugs included benzodiazepines and hypnotics, serotonin reuptake inhibitors, and neuroleptics. ASM include levetiracetam, sodium valproate, lacosamide, lamotrigine, carbamazepine, oxcarbazepine, eslicarbazepine, topiramate, phenobarbital, ethosuximide, gabapentine, zonisamide, phenytoin, stiripentol, clobazam, and clonazepam. Broad spectrum ASM used for SE treatment were levetiracetam, lacosamide, and sodium valproate in the majority of the cases and perampanel and oxcarbazepine occasionally. Cured epilepsy is the absence of seizures in the absence of any treatment for over 5 years. Alpha = 0.05/90 = 0.0006.

# Fisher's test, ^∧^Wilcoxon test, or otherwise chi-square test.

### 3.3. A higher frequency of acute brain lesions on imagery in NOSE

*Acute brain lesions on imagery* were significantly more frequent in the NOSE group (n = 10, *p* < 0.0001, and alpha = 0.0006). This included three severe traumatic brain injuries, three infectious diseases (pneumococcal meningitis, HSV1 herpes meningoencephalitis, and empyema with extensive cerebral venous thrombosis), 1 posterior reversible encephalopathy syndrome, 1 inflammatory cerebral amyloid angiopathy, 1 junctional ischemic stroke (M1 stenosis), and 1 undetermined meningoencephalitis leading to NORSE. Cases of acute brain etiologies in NISE patients were 2 severe head traumas (one of which was due to acute alcohol intoxication) and 1 ischemic stroke during meningioma surgery.

*Progressive etiologies* were all already known before SE in the NISE group and included a large majority of brain tumors (5 glioblastomas, 3 meningiomas, 2 brain metastases, and 1 cerebral lymphoma) and 1 patient with Alzheimer's disease and cerebral amyloid angiopathy, whereas 5 of 7 were discovered during SE evaluation in the NOSE group (2 glioblastomas, 1 brain metastases due to small cell lung cancer, 1 brain lymphoma recurrence, 1 cerebral cavernoma, and 2 cases of Alzheimer's disease).

*Remote brain lesions* were mainly post-traumatic and of a vascular, ischemic, or hemorrhagic nature.

### 3.4. Other heterogeneous acute factors that trigger NOSE and NISE

Other acute triggers could be associated and included forgetting ASM for 13 NISE patients (20.6%), sleep deprivation (4 NISE patients), stress (2 NISE patients), fever/sepsis (5 NOSE and 8 NISE patients), and drugs that lower the epileptic threshold (4 NOSE and 6 NISE patients). Among the 4 patients in the NOSE group with no etiology found at the time of SE, two were chronically heavy consumers of cannabis.

### 3.5. Beyond novelty or a history of epilepsy, a different expression of NOSE and NISE

NOSE tended to be diagnosed later, with a maximal delay in the diagnosis of 15 days (vs. 30 h for NISE, *p* = 0.06, alpha = 0.0006) and a median of 60 min for NOSE vs. 10 min for NISE, resulting in diagnostic and therapeutic delays (*p* = 0.006 and *p* = 0.09, respectively, alpha = 0.0006) (Table 1). SE duration was heterogeneous: on average 62 h for NOSE vs. 23 h for NISE (*p* = 0.33). In both groups, SE lasted ≥24 h in one-third of the patients and if associated with severe complications required resuscitation management in 35% of the cases. The mean duration of hospitalization was 13 days for the NOSE group [min = 4, max = 96, median = 10 days] and 10 days for the NISE group [min = 1, max = 117, median = 7 days]. Progression to refractory SE was not significantly different between the groups (*p* = 0.93).

The following heterogeneous types of SE were encountered in both NOSE and NISE: generalized convulsive, focal convulsive, initially non-convulsive, or secondary generalized convulsive in similar proportions (Table 1). However, secondary non-convulsive SE tended to be more prevalent in NOSE than in NISE (21.7% in NOSE, 6% in NISE, *p* = 0.02, alpha = 0.0006). NOSE patients tended to have more post-ictal focal neurological deficits and a greater number of severe complications, especially hemodynamic complications (*p* = 0.04 and alpha = 0.0006).

SE severity was significantly higher in NOSE than in NISE. STESS and EMSE scores were above the poor prognosis threshold in 78% of NOSE vs. 36.5% of NISE patients (36/46 ≥ 3/6 vs. 23/63; *p* < 0.0001; alpha = 0.0006) and 63% vs. 28.5% (29/46 ≥ 64/255 vs. 18/63; *p* < 0.0001; and alpha = 0.0006), respectively.

### 3.6. Different proportions of poor outcomes between baseline and the last follow-up in NOSE and NISE

**Figure 2:**
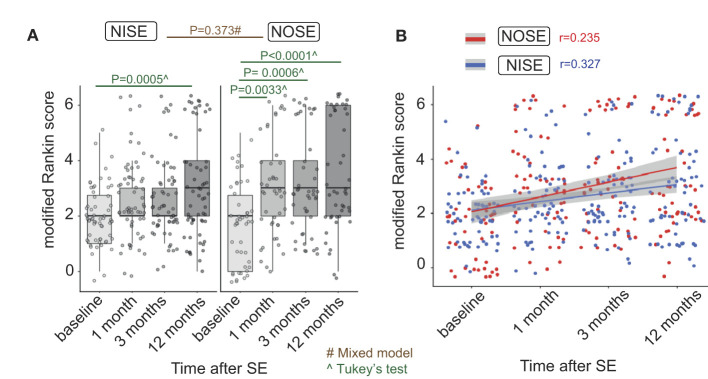
Outcome assessed by mRS between baseline and 12 months changes in different proportions in NOSE and NISE. **(A)** Individual mRS at each time point in NOSE and NISE groups. NISE patients had an average mRS of 2.02 at baseline, 2.6 at 1 month, 2.6 at 3 months, and 3.06 at 12 months. In contrast, NOSE patients had an average mRS of 2.02 at baseline, 3.3 at 1 month, 3.47 at 3 months, and 3.78 at 12 months. Therefore, we performed *post-hoc* analyses on these two groups independently. **(B)** Correlations between time after SE and outcome (Pearson correlations, *r* = 0.376 in the PMA group, *r* = 0.252 in the non-PMA group). Alpha = 0.05/3 = 0.017.

**Figure 3:**
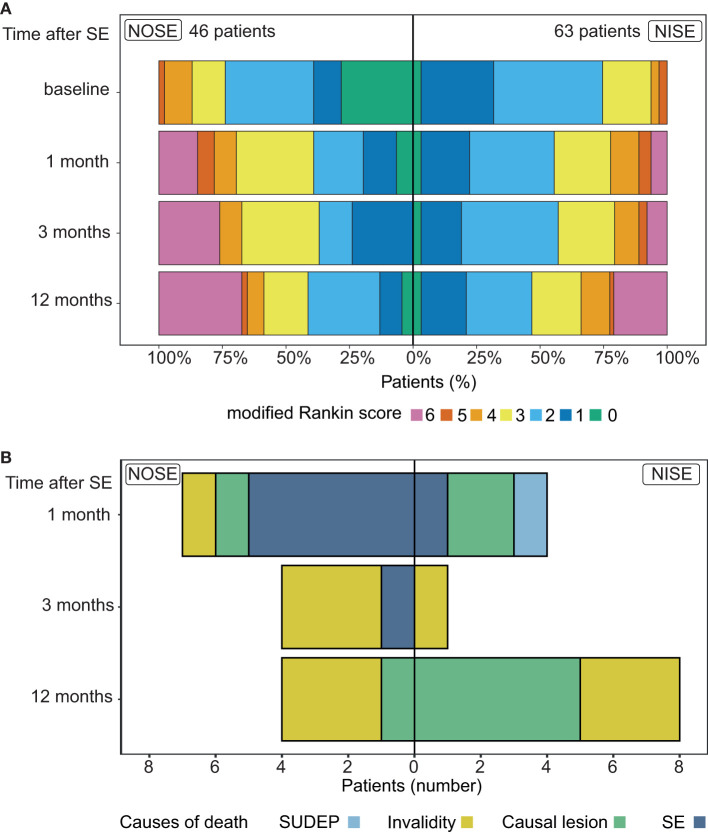
Outcome and causes of mortality in NOSE and NISE. **(A)** Outcome (mRS) changes in each group at each time point. Proportions of patients in NOSE and NISE groups for each mRS value. **(B)** Causes of mortality at each time point in NOSE and NISE groups.

There was no global effect of the inaugural or non-inaugural nature of SE on the outcome (*p* = 0.373) according to a mixed-effects model. Nevertheless, there was an interaction between the type of SE and the mRS at baseline, and in 1, 3, and 12 months (*p* < 0.017). This suggests that mRS between baseline and 12 months changes at a different speed between the two groups of patients (Figures 2, 3). For NISE patients, mRS at baseline was only different from the mRS at 12 months (Tukey's HSD test, diff = 1.05, and *p* = 0.0005). For NOSE patients, the mRS at baseline was different from the mRS at 1 month (Tukey's HSD test; diff = 1.28; and *p* = 0.0033), 3 months (Tukey's HSD test, diff = 1.46, and *p* = 0.0006), and 12 months (Tukey's HSD test, diff = 1.76; and *p* = 0).

Finally, in NOSE and NISE combined, we observed positive correlations between outcomes at the final follow-up (mRS at 12 months) and in descending order on both the STESS (Pearson correlations, *r* = 0.455, *p* = 1^−06^, and alpha <0.017) and EMSE scores (Pearson correlations, *r* = 0.326, *p* = 0.0006, and alpha <0.017) as well as the duration of SE (Pearson correlations, *r* = 0.217, *p* = 0.002, and alpha <0.017).

There was a recurrence of SE in 13 patients with NOSE (28.2%) and in 15 patients with NISE (23.8%) at 1 year but at different times: there was a recurrence in the first month in 7 cases of NOSE but only in 1 case of NISE (Fisher test, *p* = 0.01). For patients who did not die during the acute phase of SE, 17 of 39 patients with NOSE developed epilepsy (43.6%) and 29 of 52 patients with NISE had a recurrence of seizures in the year following SE (55.7%, data lacking for 11 patients). In survivors at 3 months, there was a cognitive complaint in 57% of the NOSE patients (57%) vs. 71% of the NISE patients and a focal (motor of language) deficit in 26% of NOSE and 43% of NISE cases.

### 3.7. Different causes of death at different time points in NOSE and NISE

At the final follow-up, mortality was observed in 32.6% of NOSE vs. 20.6% of NISE patients (*p* = 0.19). However, rates of death varied according to time points (Figure 3). This occurred within the first month in 15% of NOSE patients and 6% of NISE patients. The cumulated rate increased to 24% and 8% at 3 months (*p* = 0.02 and alpha = 0.0006; Table 1). The patients who died in 1 year all had refractory SE except for 1 NOSE and 2 NISE cases (25 refractory SE/28 deaths, 89%). The causes of death were diverse and variable according to the time.

In the NISE group, at 1 month, death was related to the direct consequence of a refractory SE (1 patient), to the causal lesion induced by SE (2 patients with glioblastomas), and to a probable SUDEP (1 patient); at 3 months to invalidity (one 90-year-old patient); and at 1 year, to brain tumors (3 patients with glioblastomas and 2 patients with brain metastases), and to invalidity (3 patients with multiple pathologies).

In the NOSE group, at 1 month, death was related to the direct consequence of SE (five 54- to 95-year-old patients with organ failure after NORSE), at 3 months, to organ failure in super-refractory SE (one 56-year-old man) and to invalidity after SE in a context of multiple pathologies (three 72- to 91-year-old patients), and at 1 year, to glioblastoma (1 patient) and to progressive invalidity (three 62- to 101-year-old patients).

### 3.8. The same proportion of refractory NOSE and NISE

We observed similar numbers of pharmacoresistant NOSE and NISE cases (62% and 61%, respectively). According to the mixed-effects model, there was no global effect of refractoriness on the outcome, but there was an interaction between refractoriness and the time of the mRS assessment. This indicates that between baseline and 12 months, mRS changed in different proportions in refractory and non-refractory SE (considering both NOSE and NISE). Refractory SE had an average mRS of 2.01 at baseline, 3.08 at 1 month, 3.24 at 3 months, and 3.60 at 12 months. Non-refractory SE had an average mRS of 2.01 at baseline, 3.08 at 1 month, 2.58 at 3 months, and 2.97 at 12 months. Considering that the time of mRS assessment had a different effect on refractory and non-refractory SE, we analyzed these two groups independently. For refractory SE, baseline mRS was different from the mRS at 1 month (diff = 1.06, *p* = 0.003), 3 months (diff = 1.24, *p* = 0.0004), and 12 months (diff = 1.6, *p* = 0). For non-refractory SE, mRS after SE was different from baseline only at 12 months (diff = 0.95, *p* = 0.002).

### 3.9. No discriminating value for peri-ictal MRI abnormalities

A total of 54 patients (30 NOSE and 24 NISE cases) had a 3T MRI during the initial hospitalization for SE (MRI performed within the first 72 h of admission in 35 patients). MRI revealed an acute brain etiology in 9 patients with NOSE (30%), none in NISE patients, and old brain lesions in 18 patients in each group (66%).

**Figure 4:**
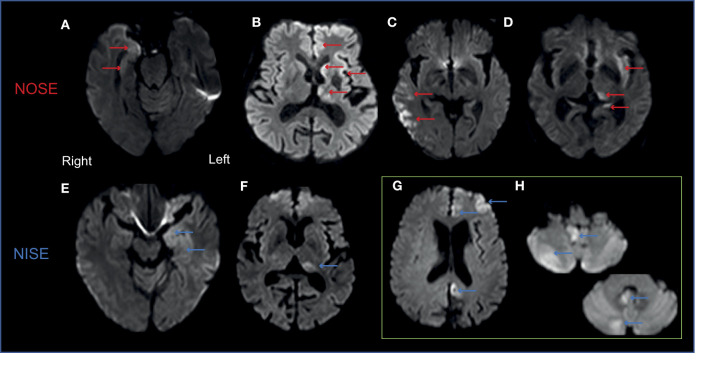
Generally similar patterns of PMA in NOSE and NISE. Examples of axial DWI views in 4 patients. **(A)** Right medial temporal hyperintensity in an 86-year-old woman with focal convulsive refractory NOSE. **(B)** Left thalamic, caudate nucleus, and frontal cortex hyperintensities in a 56-year-old man with generalized convulsive secondary non-convulsive super-refractory NOSE. **(C)** Right temporo-parietal cortex hyperintensity in a 79-year-old man with refractory generalized convulsive secondary non-convulsive NOSE. **(D)** Claustrum hyperintensity in the left hemisphere associated with a homolateral pulvinar and hippocampal hyperintensity in a 69-year-old alcoholic male with pharmacosensitive non-convulsive NOSE (SESA). **(E)** Left medial temporal hyperintensity in a 48-year-old man with pharmacosensitive focal non-convulsive NISE. **(F)** Left pulvinar hyperintensity in a 70-year-old man with pharmacosensitive focal non-convulsive NISE. **(G, H)** Right cerebellum hyperintensity and contralateral cortical hyperintensity in a 63-year-old man with a non-convulsive super-refractory NISE.

**Figure 5:**
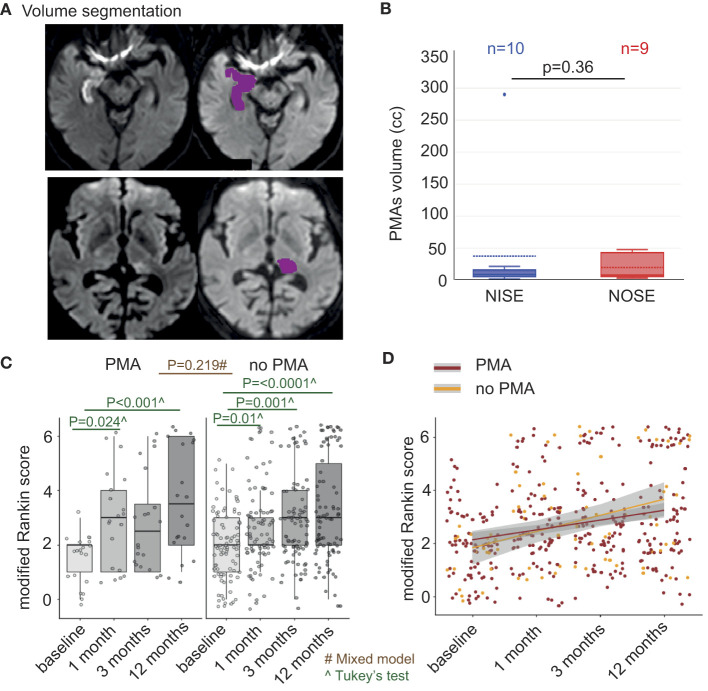
PMA volumes are similar in NOSE and NISE patients and PMA is related to prognosis. **(A)** PMA volumetry in DWI sequences. Semi-automatic quantification in one-shot analysis by OLEA (PMA is circumscribed in purple; colors were changed for purposes concerning the figure). Right mesial temporal hypersignal including the hippocampus (upper panel) and left posterior thalamic hypersignal (bottom panel). **(B)** Mean PMA volumes in each group. The two groups had a similar PMA volume (*p* = 0.36); note that there was an outlier in the NISE group with a much higher volume than the others at 290 ml, with a hypersignal in DWI of the majority of the left hemisphere cortex, the thalamus and at 7 days a right cerebellar diaschisis of partial NCSE secondarily generalized in a 52-year-old man institutionalized for encephalopathy evolving since childhood. **(C)** Individual mRS in patients with PMA and those without PMA. Patients with PMA had an average mRS of 1.5 at baseline, 3.1 at 1 month, 2.8 at 3 months, and 3.6 at 12 months. Patients without PMA had an average mRS of 1.5 at baseline, 2.2 at 1 month, 2.8 at 3 months, and 2.7 at 12 months. **(D)** Correlations between PMA evolution and outcome (mRS) at different time points (Pearson correlations, *r* = 0.376 in the PMA group, *r* = 0.252 in the non-PMA group). Alpha = 0.05/3 = 0.017.

PMAs were demonstrated in 20 patients (incidence of 37%, 10 patients in each group; Figures 1, 4, 5A). PMAs were present in 5 NOSE patients who responded to ASM, 5 NORSE, 6 refractory NISE, and 4 non-refractory NISE patients who experienced prolonged SE (180, 240, 700, and 2,000 min, respectively). The distribution of PMA and their aspect on the different MRI sequences were comparable in the 2 groups (Figure 4, Table 2). We noted the following two exceptions: a temporal punctiform gadolinium enhancement in two patients with non-refractory NOSE and a hyperintensity of the claustrum in one NOSE patient corresponding to SESA (Figure 4). There was no significant difference in PMA volume (Figure 5B, *p* = 0.36) between the 2 groups, but there was an outlier in the NISE group with a much higher volume than the others at 290 cc.

**Table 2:** MRI results in NOSE and NISE groups.

	**NOSE**	**NISE**	***p***
	***n*** = **30 (65%)**	***n*** = **24 (38%)**	**Chi**^2^ **test**
Time of MRI in relation to SE			
Peri-ictal	11	9	1
Postictal	15	12	0.82
Remission of epileptic symptoms	5	3	1^#^
Within 72 h after admission	23 (77%)	11 (46%)	0.04
Acute lesions (related to SE) visible on MRI, other than PMA	9 (30%)	0	0.01
Old brain lesions (anterior to SE) visible on MRI, other than PMA	18 (56%)	18 (75%)	0.38
PMA visible on MRI	10 (33%)	10 (42%)	0.53
MRI sequences allowing PMA visualization			
Diffusion hyperintensity	9	10	1^#^
ADC restriction	8	7	1^#^
FLAIR hyperintensity	8	8	1^#^
Gadolinium contrast enhancement	2	0	0.47^#^
Perfusion increase	3/12 (2 without PMA)	4/18 (1 without PMA)	1^#^
PMA location			
Cortex	9	4	0.057^#^
Hippocampus	8	6	0.63^#^
Amygdala	7	3	0.18^#^
Thalamus	3	4	1^#^
Pulvinar	3	3	1^#^
Claustrum	1	0	1^#^
Crossed cerebellar diaschisis	0	2	0.47^#^
Average PMA volume in cc (min-max)	20 (3.2–47.6)	31.4 (1.9–290)	0.36^∧^

PMA, peri-ictal MRI abnormalities.

# Fisher's test; ^∧^Wilcoxon test, or otherwise chi-square test. Alpha = 0.05/90 = 0.0006.

### 3.10. Relationship between PMA and outcome

According to the mixed-effects model for NOSE and NISE combined, there was no global effect of the occurrence of PMA on the outcome (*p* = 0.22). Nevertheless, there was an interaction between the occurrence of PMA and the moment of mRS assessment (baseline, at 1, 3, and 12 months; *p* = 0.000), which suggests that between baseline and 12 months, mRS changed in different proportions in the two groups of patients (Figure 5C). We analyzed PMA and non-PMA groups independently using Tukey's HSD tests. For patients with PMA, the mRS at baseline was different from the mRS at 1 month (diff = 1.55 and *p* = 0.02) and at 12 months (diff = 2.1 and *p* = 0.001). In patients without PMA, mRS at baseline was different from mRS at 1 month (diff = 0.73 and *p* = 0.01), 3 months (diff = 0.89 and *p* = 0.001), and 12 months (diff = 1.18 and *p* = 0) (Figure 5D).

Concerning PMA changes during follow-up (7 of 10 patients with PMA in each group had an MRI control at 3 months), we noted a complete regression of PMA in only 5 patients (2 NOSE and 3 NISE cases) despite systematic normalization of the DWI due to the persistence of FLAIR hyperintensity in 8 patients (4 NOSE and 4 NISE cases) and the appearance of focal atrophy in 7 patients (5 NOSE and 2 NISE cases).

### 3.11. EEG patterns of periodic lateral discharges are more prevalent in NOSE

**Figure 6:**
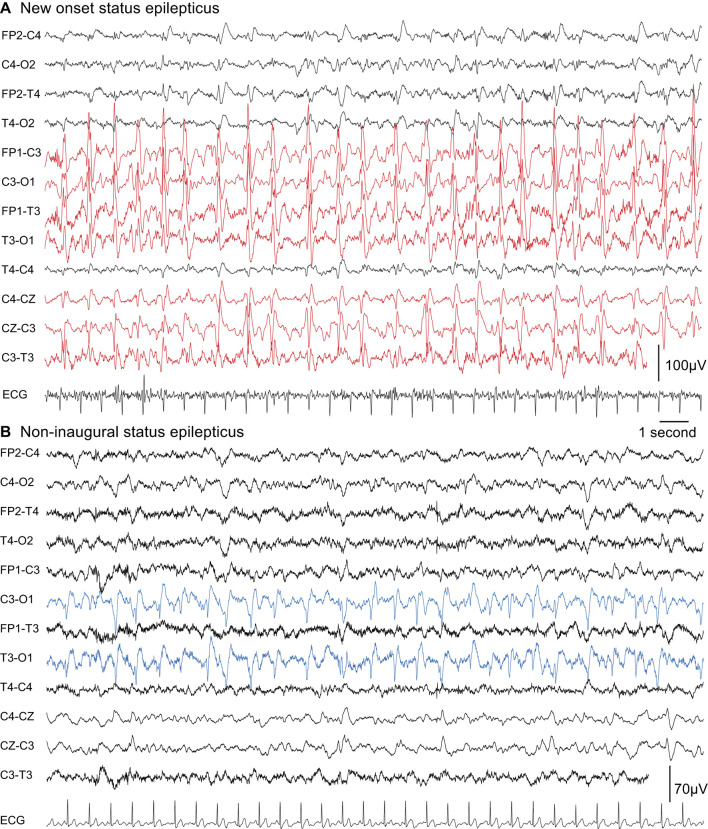
Two examples of EEG patterns that can be observed in NOSE and NISE. PLDs could be observed in the two types of SE but were more prevalent in NOSE. Here on the left hemisphere. **(A)** NOSE in a 56-year-old man (chronic psychiatric disorders and alcoholism with decompensated cirrhosis, left cerebral middle artery ischemic sequelae) who presented with a generalized convulsive SE, which became secondarily non-convulsive and super-refractory (SE lasted 41 days until death). PMAs were observed on MRI on the left hemisphere. **(B)** NISE in a 63-year-old man who presented with refractory non-convulsive SE (SE lasted 8 days). A context of stroke sequelae in the left middle cerebral artery region due to atheroma, alcoholism, and urinary tract infection. PMA was observed on MRI. After an initial stay of 54 days, there was an SE recurrence in 80 days leading to death in 4 months.

The median time before the first EEG was similar in both groups (~17 h). Different types of ictal abnormalities recorded on EEG are summarized in Table 3, and examples are provided in Figure 6. We analyzed spikes, spike-and-wave patterns, rhythmic delta discharges, and periodic epileptiform activities without identifying any specific type of epileptic activity that could be related to either NOSE or NISE. However, periodic lateralized activities tended to be more frequent in NOSE (*p* = 0.004 and alpha = 0.0006).

**Table 3:** EEG patterns during NOSE and NISE.

	**NOSE**	**NISE**	***p***
	***n*** = **46**	***n*** = **62**	
Median time before 1st EEG	17 h	17 h	0.89^∧^
Average time before 1st EEG (min-max)	39 h (3 h−384 h)	29 h (2 h−360 h)	
1st EEG conclusions			
Ictal	17 (37%)	25 (40%)	0.77
Postictal	22 (48%)	29 (47%)	0.85
Neurosedation	6 (13%)	4 (6.5%)	0.32^#^
Normal	1 (2%)	4 (6.5%)	0.39^#^
Epileptic and other pathological activities			
Epileptic discharges	14 (30%)	21 (34%)	0.74
Paroxysmal abnormalities	18 (39.1%)	23 (37%)	0.94
Spikes	15	14	0.23
Spike-and-wave patterns	5	16	0.06
Rhythmic delta discharges	10 (21.8%)	14 (22.6%)	0.96
Periodic activities	18 (39.1%)	8 (13%)	0.003
PLD	17	8	0.004
GPD	1	0	0.43^#^
Burst suppressions	0	2 (3.2%)	0.51^#^
EEG leading to SE diagnosis			
Generalized convulsive	0	1 (1.6%)	0.87^#^
Focal convulsive	5 (10.8%)	3 (4.8%)	0.40
Non-convulsive	12 (26%)	8 (13%)	0.12
Mean number of EEGs after the 1st one (median; min–max)	2 (1; 0–10)	2 (3; 0–16)	0.85
Long-term EEG monitoring	6 (13%)	6 (9.7%)	0.79

# Fisher's test; ^∧^Wilcoxon test, otherwise chi-square test. PLD, periodic lateralized discharges; GPD, generalized periodic discharges.

Alpha = 0.05/90 = 0.0006.

## 4. Discussion

Our study reveals a paradox: NOSE was more severe with more patients experiencing a poor outcome at the last follow-up but was not more refractory than NISE. Causes of death also differed at different time points of follow-up, with more early deaths directly linked to SE at 1 month in the NOSE group and more remote deaths related to causal brain lesions at the final follow-up in the NISE group.

We are aware of the limitations of our study: the clinical heterogeneity of the patients, heterogeneous types of SE and etiologies, outliers, MRI performed only in approximately half of the patients, and the difficulty assessing the kinetics of PMA. However, such limits are inherent in this type of prospective research precisely because of the severity of SE that can limit inclusion. Including more than 100 patients is rare and we were able to provide longitudinal data, without any patients lost to follow-up. Moreover, when available, MRI was always performed at a high resolution (3T) and within relatively homogeneous time frames.

### 4.1. NOSE patients are older than NISE patients at the onset

There is an undeniable fragility in NOSE patients at the onset: 61% had a preexisting cognitive decline (IQ Code ≥ 3.4). This fragility was not limited to the NOSE group: two-thirds of the patients had a significant disability (mRS ≥ 2) in both groups. The mean age of the patients in our study was 60 years, similar to the ages in previous cohorts: 60–65 years according to the cohort (15, 17, 23, 24, 45–47). However, NOSE generally occurs in older subjects (65 years vs. 56 years in NISE patients). Therefore, it is interesting to note that epileptic and non-epileptic patients were comparable in the literature for neurological and psychiatric history, previous psychotropic drug use, and alcohol abuse or dependence (16, 17, 24, 48). In particular, SESA was not overrepresented in NOSE.

### 4.2. NOSE is not more refractory than NISE but tends to be more severe

In both groups, SE was long, lasting ≥24 h in one-third of our patients, and if associated with severe complications required resuscitation management in 35% of the cases. This is consistent with previous studies in which the duration of SE was ≥ 24 h in 24–33% of the cases (15, 16) and only 7% of SE lasted <30 min (45).

In our study, the proportions of pharmacoresistance in SE (more than 60% of the cases in both groups) contrast with the 20–40% of refractoriness reported in the literature (16, 17, 45, 48, 49), although we used the same definitions of drug resistance and clinical management according to the national recommendations for therapeutic escalation (33).

However, as in the literature, NOSE was more severe than NISE. Indeed, the severity scales of NOSE were higher (*p* < 0.001), the duration was longer (62 vs. 23 h on average), and the diagnostic tardiness was greater (60 vs. 10 min) resulting in a therapeutic delay. NOSE patients had more post-ictal focal neurological deficits (80% vs. 62% in NISE) and more serious complications (50% vs. 30% in NISE), especially hemodynamic complications. The length of hospital stay was also longer for NOSE patients than for NISE patients, on average 3 days. This is in line with the previously identified prognostic factors of SE, such as the age of the patient, the rapidity of the diagnosis and suitable therapeutic management, and the duration and etiology of SE (15–17, 19, 24, 47, 50, 51).

### 4.3. NOSE becomes secondarily non-convulsive more often than NISE

In our cohort, 36.7% of the SE were non-convulsive (NCSE), whereas NCSE accounts for 25% of the SE in the literature (21, 22, 52). NCSE is frequent (up to 47% of the SE managed in the Intensive Care Unit (53, 54) but is underestimated due to the misleading and non-specific clinical features. In fact, the diagnosis is mainly based on the EEG, and quite frequently there is a diagnostic delay and high pharmacoresistance in nearly one-third of the cases (55–58). NCSE has been associated with a longer duration and a worse prognosis than the other subtypes of SE, especially when it is inaugural because of its frequent refractoriness (50). The mortality rate of the inaugural NCSE [nearly 69% of NCSE in some cohorts (58)] can be as high as 40%, whereas it is ~10% for the other SE (59).

### 4.4. In line with novelty, more acute brain lesions occur in NOSE

In a recent retrospective cohort including 85 NOSE patients, the main etiologies were acute symptomatic NOSE in 53.9%, unknown in 25.9%, progressive in 11.8%, and remote in 9.4%. For adults below the age of 60 years, the main etiology remained unknown (36.3%) followed by autoimmune-related SE (16.4%), while in the elderly (≥60 years), the primary etiology was central nervous system infection (23.3%) followed by cerebrovascular disease (20%) and intracranial tumors (20%) (23). In the 89 patients reported by Santamarina et al. (mean age of 69 years), NOSE had an acute etiology for 66.3% of the patients (46.1% brain lesions and 20.2% toxic/metabolic causes), a remote or progressive etiology for 19.1% of the patients, and remained cryptogenic for 14.6% of the patients (27).

If the etiologies overlap with these references, we emphasize different proportions according to these causes in our cohort (perhaps related to the fact that our NOSE cohort was smaller). The etiology of NOSE was acute in 43.5% of the cases vs. 8% in NISE (*p* < 0.0001), including an acute cerebral etiology in 21.7% of NOSE vs. 4.8% of NISE cases (*p* = 0.01). The etiology remained unknown in only 8.7% of the NOSE cases. However, by systematically comparing them to NISE, our results highlight the etiologies shared by both types of SE. For instance, we observed no autoimmune encephalitis, whereas this was the etiology for epilepsy in two NISE patients. We also noted 3 cases of non-convulsive NOSE that met the subacute encephalopathy and seizures in alcoholics (SESA) criteria (5, 60, 61), all with PMA on ictal MRI.

### 4.5. Poor outcome between baseline and last follow-up is more frequent in NOSE

To the best of our knowledge, the only study that has prospectively compared NOSE and NISE was restricted to 122 patients >60 years old with convulsive SE. It showed that comorbidities, a low Glasgow scale score, and an inaugural nature were poor prognostic factors (62). By including younger subjects and all types of SE, we were able to demonstrate the frailty and older age of NOSE subjects.

We also observed that the mRS increased at each assessment time in both groups. However, after *post-hoc* analyses, baseline mRS (before SE) for NOSE was statistically different from mRS at 1, 3, and 12 months, while for NISE, it was only different from mRS at 12 months.

In our cohort, 43.6% of the NOSE patients who survived developed epilepsy in the ensuing months. For Santamarina et al., it was close to 58.7% (27), which highlights the relevance of long-term maintenance of an ASM after SE. A total of 25.7% of patients had a recurrence of SE during follow-up, including 28.2% NOSE cases [twice the rate found in a previous prospective cohort (45)]. There were more early recurrences of SE in NOSE patients (*p* = 0.01), probably associated with difficulty in controlling the initial SE and the underlying etiology.

We noted a high frequency of focal neurological deficits (34.4%) and cognitive complaints (70%) in both groups at 3 months but which were higher for NISE. Cognitive consequences are frequently reported in the literature and have a significant impact on quality of life (50, 63, 64). Therefore, in the clinical management of any SE, it would be relevant to conduct psychometric and standardized cognitive assessment some distance in time after NOSE and NISE to propose cognitive remediation adapted to these fragile patients.

### 4.6. Different causes of remote and early deaths in NOSE and NISE

The overall mortality of our population was high: 10%, 17%, and 26% at 1, 3, and 12 months, respectively. These rates are comparable to the mortality found in cohorts with any type of SE (NOSE and NISE, excluding post-anoxic encephalopathies) (17, 48) or even studies including only refractory SE with 24.5–25.4% mortality at 1 year (38, 65). Similarities to these rates can probably be explained by the high drug resistance in our cohort. In specific NORSE cohorts, mortality reached 22% (3, 28).

In our study, a higher global proportion of death was observed in NOSE than in NISE. The absence of statistical difference at the final follow-up does not exclude differences at distant time points and may reflect different mechanisms: more early deaths in the NOSE group directly linked to SE at 1 month and more remote deaths linked to causal brain lesions in the NISE group at final follow-up. In addition, it has been previously shown that NOSE is associated with a 15-fold increase in the risk of death in those older than 60 years (66).

### 4.7. For all SE in general, patients with PMA have a poorer prognosis at the last follow-up

The description and location of PMA in our cohort were comparable to previously published data (67–71): DWI hyperintensity with a moderate ADC restriction and FLAIR hyperintensity, preferentially in the hippocampus, the cortex, the amygdala, and the thalamus. In our cohort, both NOSE and NISE had PMAs that were comparable in appearance, location, and volume. These results are important because contrary to common belief, PMAs are not exclusive to NORSE and in general and are not exclusive to NOSE. The overall incidence of PMAs (37%) is close to the incidences found in studies that did not consider only abnormal MRI: 27.5% (25), 28% (72), and 42.5% (67). The incidence of PMAs was somewhat higher in the NISE group (42% vs. 33%) but their MRIs were performed later than those for NOSE patients. This difference could be due to the kinetics of the occurrence of PMAs.

A significant correlation has already been described between the presence of PMAs and the presence of PLDs on EEG with vigilance in the acute phase of SE, but no association was found with patient age, comorbidity, or mortality (25). Our clinical experience suggests that there is a very good anatomical correlation between the clinical symptoms of SE, the site of EEG abnormalities, and the location of PMA when present. Post-ictal motor deficits have also been more frequently associated with PMAs (53.3%) than with normal MRI (34.4%) (67). In our data, the outcome at the last follow-up changed in different proportions in the patients with PMA and those without PMA, while no correlation exists between PMA volume and mRS during follow-up. There does not appear to be any specific feature (volume, locations, and change) of PMAs that can distinguish a peri-ictal from a post-ictal state. Rapid brain imagery is recommended in the etiological assessment of any SE (10, 33). Our results highlight that in addition to the diagnostic potential and the identification of acute lesions, MRI provides prognostic information that may prove valuable in long-term patient management.

In the neuroimaging studies cited above, MRI monitoring was not systematic and completion time was extremely variable (interval of up to 1 year between the two MRIs) (68–70). In our study, MRI time was more homogeneous at ~3 months and complete reversibility of PMA was noted in 36% of patients. Although diffusion was normalized, FLAIR hyperintensity was found in 57% of patients and focal atrophy in 50% of patients, predominantly in the temporal-hippocampal regions. These sequelae raise questions about the epileptogenic value and the clinical consequences of these permanent structural abnormalities.

### 4.8. Periodic lateral discharges are more frequent in NOSE than in NISE

We observed a tendency for more periodic lateral discharges (PLDs) in NOSE, while the median time before the first EEG was similar in both groups. Despite a later diagnosis in NOSE, accessibility to EEG was not different for NOSE and NISE, which is a crucial point to remember in clinical practice. PLDs were indicative of the presence of a cortical brain lesion and associated with more frequent vigilance disorders as previously described (73). PLDs were also associated with high morbidity and mortality in studies conducted in the eighties or nineties (74–76). We were unable to determine whether there is a PLD pattern (morphology, periodicity, and amplitude) specific to each type of SE. Further studies that analyze the appearance of PLDs according to the etiology, lesion, and type of SE are warranted.

### 4.9. NORSE vs. non-inaugural refractory SE

NOSE was not more refractory than NISE. Unfortunately, our data were not sufficient to highlight specificities between the particular cases of NORSE and non-inaugural refractory SE (NIRSE).

Are there certain etiologies that are particularly represented in refractory SE? In other series that focused specifically on the NORSE subgroup, the most common etiology was autoimmune encephalitis, while 52% of the cases of NORSE remained cryptogenic (3, 28, 31, 77). No autoimmune encephalitis was identified in our NOSE cohort although this etiology was repeatedly suspected and sought, while two cases of autoimmune encephalitis were included in our NISE group with a refractory SE. Two patients in our cohort met the criteria for cryptogenic NORSE: the first died within a few days despite appropriate resuscitative management while the second patient had severe cognitive impairment and loss of autonomy at 3 months.

Moreover, our data were not sufficient to isolate specific patterns of PMA between NOSE and NORSE. However, particularities in three patients should be noted: a case of claustrum hyperintensity in the NOSE group corresponding to SESA with non-refractory NCSE. In a recent study, claustral changes were reported as infrequent, occurring in 9.1% of NORSE patients (78) although the etiology for NORSE patients with claustrum involvement has not yet been elucidated. The claustrum sign has been associated with an aggressive refractory form of SE in particular cases of FIRES with cryptogenic etiology (78, 79) but never with SESA as far as we know. Two NISE patients presented with crossed cerebellar diaschisis with involvement of the cortex and the pulvinar ipsilateral to the refractory SE. Some identical cases have been described in the literature with reversible damage or the appearance of cerebellar atrophy associated with an unfavorable clinical course (80–84).

## 5. Conclusion

NOSE and NISE evolved in the same proportions as refractory SE and shared common patterns, such as the same types of PMAs on MRI. However, NOSE was distinguishable by the severity, a more fragile and older population at onset, and a frequent non-convulsive semiology. The causes of death differed in the early and late stages (at 1 year) in NOSE and NISE. Despite acute causal brain lesions, the inaugural character was still too often associated with a delay in diagnosis in SE, which justifies the need to more clearly specify the types of SE to constantly raise awareness among clinicians. These results also highlight the relevance of including novelty-related criteria, clinical history, and the temporality of occurrence in the nosology of status epilepticus.

## Data availability statement

The raw data supporting the conclusions of this article will be made available by the authors, without undue reservation.

## Ethics statement

The studies involving human participants were reviewed and approved by Regional Ethics Committee at Toulouse University Hospital (CPP Sud-Ouest no. 04-1215). Written informed consent for participation was not required for this study in accordance with the national legislation and the institutional requirements.

## Author contributions

MB and JC: writing of the manuscript, major role in data acquisition and study design, data analysis and interpretation, and expert analysis of EEG. LV: revision of the manuscript, expert analysis of EEG, data analysis and interpretation, major role in the study design, and help with data acquisition. MD, FR, and RD: expert analysis of EEG and revision of the manuscript. EB: revision of the manuscript, data analysis, and interpretation. FB: expert analysis of MRI, major role in study design, and help with data acquisition. LG: statistical analyses and revision of the manuscript. VW: support for figures and statistical analyses. All authors contributed to the article and approved the submitted version.
